# Phase II, open-label, multicenter trial of crizotinib in Japanese patients with advanced non-small cell lung cancer harboring a MET gene alteration: Co-MET study

**DOI:** 10.1186/s13063-020-4221-7

**Published:** 2020-03-30

**Authors:** Mototsugu Shimokawa, Kaname Nosaki, Takashi Seto, Kadoaki Ohashi, Masahiro Morise, Hidehito Horinouchi, Jun Sakakibara, Haruyasu Murakami, Seiji Yano, Miyako Satouchi, Shingo Matsumoto, Koichi Goto, Kiyotaka Yoh

**Affiliations:** 1 Cancer Biostatistics Laboratory, Clinical Research Institute, National Hospital Organization Kyushu Cancer Center, Fukuoka, Japan; 2 Department of Biostatistics, Yamaguchi University Graduate School of Medicine, 1-1-1 Minamikogushi, Ube, Yamaguchi 755-8505 Japan; 3 Department of Thoracic Oncology, National Hospital Organization Kyushu Cancer Center, Fukuoka, Japan; 4 Department of Thoracic Oncology, National Cancer Center Hospital East, Chiba, Japan; 5 Department of Allergy and Respiratory Medicine, Okayama University Hospital, Okayama, Japan; 6 Department of Respiratory Medicine, Nagoya University Graduate School of Medicine, Nagoya, Japan; 7 Department of Thoracic Oncology, National Cancer Center Hospital, Tokyo, Japan; 8 First Department of Medicine, Hokkaido University Hospital, Hokkaido, Japan; 9 Division of Thoracic Oncology, Shizuoka Cancer Center, Shizuoka, Japan; 10 Division of Medical Oncology, Cancer Research Institute, Kanazawa University, Kanazawa, Japan; 11 Department of Thoracic Oncology, Hyogo Cancer Center, Hyogo, Japan

**Keywords:** Non-small cell lung cancer, Crizotinib, MET gene alteration, RT-PCR assay, Next-generation sequencing

## Abstract

**Background:**

MET-deregulated non-small cell lung cancer represents an urgent clinical need because of the lack of specific therapies. Although recent studies have suggested a potential role for crizotinib in patients harboring MET gene alterations, no conclusive data are currently available. Therefore, we designed the Co-MET study, a single-arm phase II study to assess the efficacy and safety of crizotinib in patients with advanced non-small cell lung cancers harboring MET gene alterations.

**Methods:**

Co-MET is an open-label, multi-center, single-arm, phase II trial to assess the safety and efficacy of oral crizotinib in patients with advanced non-small cell lung cancer harboring MET exon 14 skipping mutation (cohort 1) or a high MET gene copy number of ≥ 7 (cohort 2). We will identify MET gene alterations using RT-PCR and/or next-generation sequencing. Oral crizotinib 250 mg BID will be administered until disease progression or unacceptable toxicity. A radiology committee will review tumor scans according to the RECIST criteria. The primary endpoint is the objective response rate. Assuming a null hypothesis of 20% objective response rate and an alternative hypothesis of 50% objective response rate for cohort 1, and a one-sided alpha error of 0.05 and 80% power based on the exact binomial distribution, the required number of evaluable patients is 19. We set the exploratory sample size for cohort 2 at 10 patients.

**Discussion:**

The results of this study are expected to provide evidence regarding the usefulness of oral crizotinib for advanced MET exon 14 skipping mutation-positive or MET high gene copy number-positive non-small cell lung cancer.

**Trial registration:**

This study was registered with the University Hospital Medical Information Network Clinical Trials Registry as UMIN000031623 on 3 March 2018.

## Background

Non-small cell lung cancer (NSCLC) is a common cause of cancer mortality worldwide. The histological diagnoses include ~ 85% of non-small cell and ~ 15% of cell lung cancers. The majority of patients with NSCLC have a metastatic disease at diagnosis, for which no curative treatment exists. Platinum-based chemotherapies were standard for patients with NSCLC and good performance status. Phase III randomized trials of tyrosine kinase inhibitor (TKI) therapy for EGFR-mutant and anaplastic lymphoma receptor tyrosine kinase (ALK)-rearranged lung cancers have shown documented improvements in response and progression-free survival (PFS) [1–3], and TKIs are approved for patients with oncogene-driver mutations. NSCLC represents a paradigm for the development of targeted cancer therapy. Advancements in next-generation sequencing (NGS) technology have greatly assisted the discovery of rare driver mutations that may serve as potential therapeutic targets in lung cancer [4, 5].

c-Met is the tyrosine kinase receptor for hepatocyte growth factor (HGF). Binding of HGF to MET stimulates downstream signal pathways, such as the RAS/ERK/MAPK, PI3K/AKT, Wnt/β-catenin, and STAT signaling pathways. These pathways are known to involve cell growth, migration, angiogenesis, and survival. MET gene alterations, including MET exon 14 skipping mutation-positive or MET high gene copy number, generate oncogenes via activation of c-MET signaling pathway [5, 6].

Crizotinib is a selective ATP-competitive small-molecule inhibitor of c-Met, ALK, and ROS1 (c-ros) tyrosine kinases. Dramatic and durable responses to crizotinib were first reported in mid-2015 in patients with advanced NSCLC harboring MET exon 14 skipping mutation [7–9]. Crizotinib also demonstrated efficacy in NSCLC with high MET gene copy number [10, 11].

Since MET-deregulated NSCLC represents an urgent clinical need because of a lack of approved specific therapies, we designed a trial to assess the efficacy and safety of crizotinib in patients with advanced NSCLCs harboring MET gene alterations. The planned protocol will include 29 response-evaluable patients with advanced NSCLC whose tumors contain MET exon 14 skipping mutations or high MET gene copy number. We will use a validated reverse transcription polymerase chain reaction (RT-PCR) and/or NGS assay (multi-screening using oncomine comprehensive assay (OCA) panel) to identify MET gene alterations.

## Methods/Design

### Study design and objective

**Fig. 1:**
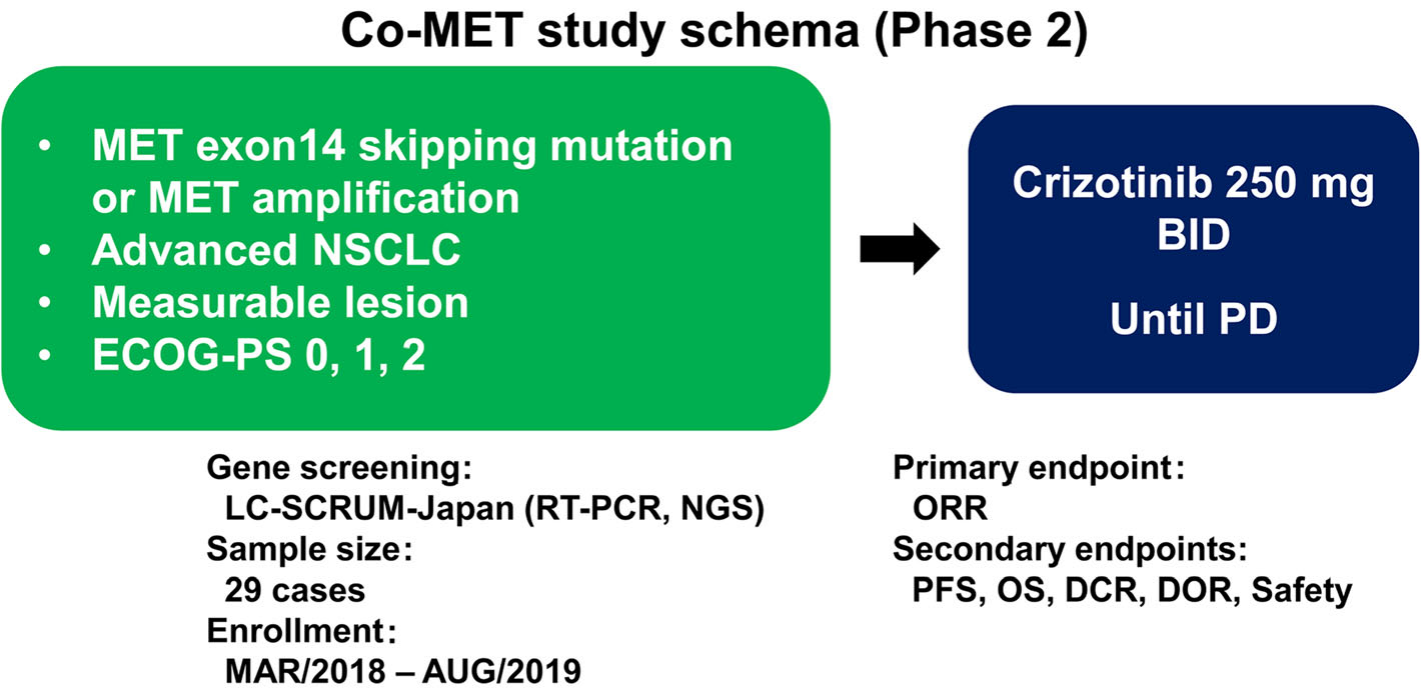
Co-MET study schema (phase 2)

This is an open-label, multi-center, two cohort, single-arm, phase 2 trial of oral crizotinib in patients with advanced NSCLC harboring MET exon 14 skipping mutation (cohort 1) or high MET gene copy numbers of seven or more (cohort 2) (Fig. 1). The population in cohort 1 includes patients with advanced NSCLC harboring MET exon 14 skipping mutation who have not received prior MET inhibitor treatments. We will confirm MET gene alterations using a validated RT-PCR and/or NGS assay. Our primary objective is to assess the antitumor activity of oral single-agent crizotinib for advanced MET exon 14 skipping mutation-positive or MET high gene copy number-positive NSCLCs as measured by the objective response rate (ORR). Our secondary objectives included assessing secondary measures of antitumor activity by duration of response (DR); disease control rate at 8, 16, and 24 weeks; and PFS and overall survival and the safety and tolerability of oral crizotinib. This study will be conducted according to the standards of Good Clinical Practice and in compliance with the principles of the Declaration of Helsinki, and our Institutional Review Board has approved the protocol registered in the University Hospital Medical Information Network Clinical Trials Registry as UMIN000031623 (https://upload.umin.ac.jp/cgi-open-bin/ctr_e/ctr_view.cgi?recptno=R000035817). In this study, academic hospitals where at least one investigator-initiated clinical trial has been conducted in accordance with GCP standards will be recruited. Therefore, this study will be conducted at National Hospital Organization Kyushu Cancer Center, Okayama University Hospital, Hyogo Cancer Center, Nagoya University Hospital, Shizuoka Cancer Center, Kanazawa University Hospital, National Cancer Center Hospital, National Cancer Center Hospital East, and Hokkaido University Hospital, Japan.

### Eligibility criteria

Table 1 shows key inclusion and exclusion criteria.

**Table 1:** Inclusion and exclusion criteria

Inclusion criteria	
1. Histologically or cytologically confirmed NSCLC that is locally advanced or metastatic	
2. Positivity for MET exon 14 skipping mutation or high MET copy number (seven or more) as determined by a validated RT-PCR and/or NGS assay by a designated central testing laboratory (LC-SCRUM)	
*Patients positive for MET exon 14 skipping mutation and high MET copy number of seven or more will be enrolled in cohort 1.	
3. At least one measurable tumor lesion as per RECIST (version 1.1) that has not been irradiated	
4. Women or men, 20 years of age or older	
5. ECOG performance status between 0 and 2	
6. Adequate organ function as defined by the following criteria:	
Serum aspartate transaminase (AST) and serum alanine transaminase (ALT) < 2.5 x upper limit of normal (ULN), or AST and ALT < 5 x ULN if liver function abnormalities are due to underlying malignancy	
Total serum bilirubin < 1.5 x ULN	
Absolute neutrophil count (ANC) > 1500/μL	
Platelets > 50,000/μL	
Hemoglobin > 8.0 g/dL	
Serum creatinine < 2 x ULN	
7. Signed and dated informed consent document indicating that the patient has been informed of all the pertinent aspects of the trial prior to enrollment	
8. Willingness and ability to comply with scheduled visits, treatment plans, laboratory tests, and other study procedures	
9. Agreement to use effective contraception during the study period and for at least 90 days after the last dose of crizotinib	
Exclusion criteria	
1. Current treatment on another therapeutic clinical trial	
2. Characterized ALK or ROS1-positive rearrangement	
3. Prior therapy specifically directed against MET	
4. Any treatment (chemotherapy, radiation, or surgery) within 2 weeks prior to study entry, except for patients who completed palliative radiation 48 h prior to study entry	
5. Any acute toxicity > Grade 1	
6. Symptomatic brain metastases. Eligible if asymptomatic, or if treated (must be neurologically stable for at least 2 weeks and are not taking unstable or increasing doses of corticosteroids)	
7. Spinal cord compression unless treated with the patient attaining good pain control and stable or recovered neurologic function, carcinomatous meningitis, or leptomeningeal disease	
8. Known interstitial fibrosis or interstitial lung disease	
9. Any of the following within the 3 months prior to starting study treatment: myocardial infarction, severe/unstable angina, coronary/peripheral artery bypass graft, congestive heart failure, or cerebrovascular accident including transient ischemic attack	
10. Ongoing cardiac dysrhythmias of NCI CTCAE v4.03 Grade > 2, uncontrolled atrial fibrillation of any grade, or QTc > 470 msec	
11. Pregnancy or breastfeeding	
12. Use of drugs or foods after study enrollment that are potent CYP3A4 inhibitors, including but not limited to atazanavir, clarithromycin, indinavir, itraconazole, ketoconazole, nefazodone, nelfinavir, ritonavir, saquinavir, telithromycin, troleandomycin, voriconazole, and grapefruit or grapefruit juice	
13. Use of drugs after study enrollment that are potent CYP3A4 inducers, including but not limited to carbamazepine, phenobarbital, phenytoin, rifabutin, rifampin, and St. John’s wort	
14. Use of drugs after study enrollment that are CYP3A4 substrates with narrow therapeutic indices, including but not limited to dihydroergotamine, ergotamine, pimozide, astemizole, cisapride, and terfenadine	
15. Any other anticancer drugs including traditional Chinese medicine are prohibited	
16. Evidence of active malignancy (other than NSCLC, non-melanoma skin cancer, localized cervical cancer, or localized and presumed cured prostate cancer) within the last 3 years	
17. Other severe acute or chronic medical or psychiatric conditions, or laboratory abnormalities that would impart, in the judgment of the investigator, excess risk associated with study participation or study drug administration, and which would, therefore, make the patient inappropriate for entry into this study	
18. Patients whom investigator judges to be inappropriate as participants	

### Patient registration

After eligibility criteria have been confirmed and informed consent has been obtained, eligible patients are registered, and the investigators initiate the planned treatment. On the consent form, participants will be asked if they agree to the use of their data should they choose to withdraw from the trial. Participants will also be asked for permission for the research team to share relevant data with people from the Universities taking part in the research or from regulatory authorities, where relevant. This trial involves collecting biological specimens for storage.

### Treatment plan

Crizotinib 250 mg BID will be administered orally at the same time on each day on a continuous daily dosing schedule unless sufficient toxicity develops to warrant dosing interruption/dose reduction. The cycles are defined as 28-day periods.

### Efficacy and safety evaluation

Table 2 lists the time points for assessments of efficacy, adverse events, laboratory safety assessments (hematology, coagulation, and chemistry), physical examination, ECG, and tumor measurements. We will perform CTs and/or MRI scans every 8 weeks, and after 12 cycles, every 12 weeks for tumor assessments.

**Table 2:**
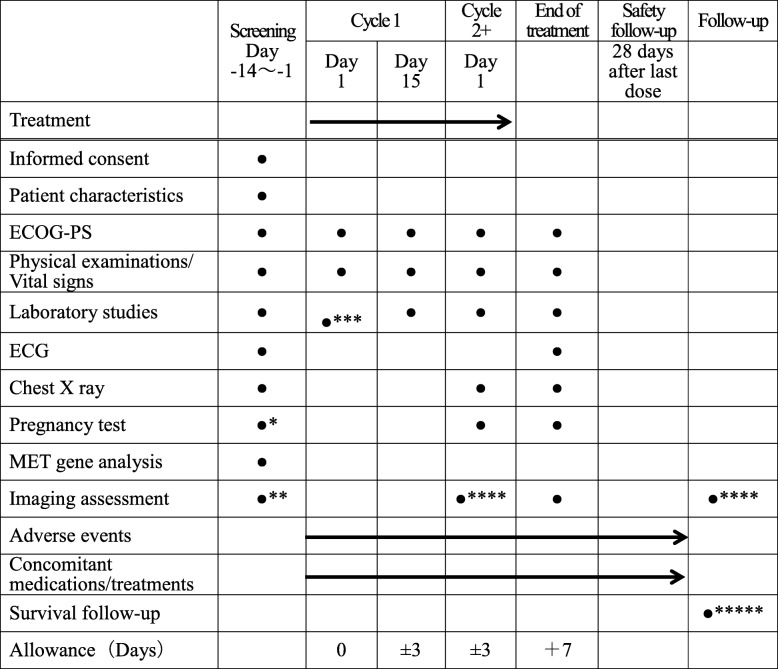
Schedule of activities

* After Cycle 1 Day 1, pregnancy test should be performed when pregnancy is suspected

** Imaging assessments (Contrast CT of chest, abdomen, and pelvis and brain CT or MRI scan with contrast) will be performed at Day −28 to Day −1 prior to Cycle 1 Day 1. Bone MRI is not required unless clinically indicated

*** Laboratory studies can be substituted with data carried out within 7 days

**** Every 8 weeks and, after 12 cycles, every 12 weeks, assessment will be performed using CT and/or MRI scan (+/− 7 days window). Chest X-rays are not required at the time of CT examination. Patients who discontinue prior to RECIST v1.1-defined progressive disease will continue with tumor assessments according to the protocol until disease progression is documented or initiation of additional anticancer therapy

***** Survival information will be collected every 3 months until death, loss to follow-up, or withdrawal of consent for survival. The investigator will collect survival information until 1 year after the last patient has enrolled into the study

### Adverse events

Adverse events will be classified based on the type, incidence, severity (graded by the National Cancer Institute Common Terminology Criteria for Adverse Events [CTCAE] Version 4.03), timing, seriousness, and relatedness. Adverse events are defined as the appearance of (or worsening of any pre-existing) undesirable sign, symptom, or medical condition that occurs after the first dose of the study treatment. Adverse event monitoring should be continued for at least 28 days following the last dose of the study treatment or until the start of subsequent antineoplastic chemotherapy.

### Data collection and management

All data will be recorded in a case report form (CRF) by investigators or clinical research coordinators. The completed CRFs will be submitted to the data center at the Five Rings Company. All study documents will be regarded as confidential. In the data center, the data will be stored and handled in a secure server maintaining the anonymity of participants.

### Statistical method

In cohort 1, an ORR of 20% is a clinically meaningful threshold for this study. We based the sample size on the exact binomial distribution. Assuming a null hypothesis of a 20% ORR and an alternative hypothesis of a 50% ORR, and a one-sided alpha error of 0.05 (one-side) and 80% power, the required evaluable patients with MET exon 14 skipping mutation-positive NSCLC is 19.

We expect cohort 2 to include mostly second- or later-line patients. The ORR of second-line single-agent chemotherapy in that clinical setting is approximately 10%. The ORRs of second- or later-line treatment with PD-1/PD-L1 inhibitor for patients previously treated with platinum-based chemotherapy are 18–20% [12–15].

We determined the sample size for cohort 2 based on the precision of ORR as a primary endpoint.

### Monitoring

An independent Data and Safety Monitoring Committee has been established to assess the safety data when serious adverse events may occur. A qualified and independent auditor is appointed to audit the trial systems, and the trial was conducted before and during the study in accordance with a written procedure.

### Post-trial care

This clinical trial will adopt clinical trial insurance; if serious harm occurs following the clinical trial, the affected participants will receive appropriate coverage.

## Discussion

MET-TKIs being developed target the MET exon 14 skipping+ NSCLCs (NCT00585195, NCT02864992, NCT02414139). With the safety data of crizotinib firmly established, we expect its imminent approval as the first MET-TKI.

A sensitive, reliable molecular test should be incorporated into the trial to obtain a compendium of approved diagnostics for MET alterations. Therefore, we allowed enrollment of patients positive for MET exon 14 skipping mutation or high MET copy number of seven or greater, as determined by a validated RT-PCR and/or NGS assay by a designated central testing laboratory (LC-SCRUM) in this trial.

If crizotinib demonstrates a meaningful clinical benefit in Japanese patients with MET exon 14 skipping mutation-positive NSCLC in this trial, our results will help crizotinib receive approval for these patients in Japan.

In conclusion, our study will evaluate the efficacy and safety of oral single-agent crizotinib for the treatment of advanced MET exon 14 skipping mutation-positive or MET high gene copy number-positive NSCLC.

### Trial status

The trial has been registered at the University Hospital Medical Information Network Clinical Trials Registry (registration number: UMIN000031623; registration date: 3 March 2018; https://www.umin.ac.jp/ctr/index.htm). Recruitment started in March 2018 and is expected to finish in May 2020. The protocol version number is 1.0.

## Data Availability

We will disseminate the results of this clinical trial widely through conference presentations and publications in relevant journals. All evaluation forms, reports, and other records will be identified in a manner designed to maintain participant confidentiality. All records will be kept in a secure storage area with limited access. The data from this clinical trial will not be shared. The Investigator and study site staff involved with this study may not disclose or use for any purpose other than performance of the study, any data, record, or other unpublished, confidential information disclosed to those individuals for the purpose of the study. The Co-MET study does not involve collecting biological specimens for storage.

## Abbreviations

ALK: Anaplastic lymphoma receptor tyrosine kinase
BID: b.i.d. oral dose
DCR: Disease control rate
DR: Duration of response
HGF: Hepatocyte growth factor
MET: Mesenchymal-epithelial transition
NGS: Next-generation sequencing
NSCLC: Non-small cell lung cancer
ORR: Objective response rate
OS: Overall survival
PFS: Progression-free survival
RECIST: Response Evaluation Criteria in Solid Tumors
TKI: Tyrosine kinase inhibitor
ULN: Upper limit of normal
RT-PCR: Reverse Transcription Polymerase Chain Reaction
OCA: Oncomine Comprehensive Assay
